# A TPMS-based method for modeling porous scaffolds for bionic bone tissue engineering

**DOI:** 10.1038/s41598-018-25750-9

**Published:** 2018-05-09

**Authors:** Jianping Shi, Liya Zhu, Lan Li, Zongan Li, Jiquan Yang, Xingsong Wang

**Affiliations:** 1 School of Mechanical Engineering, Southeast University, Nanjing, 211189 China; 2 Jiangsu Key Laboratory of 3D Printing Equipment and Manufacturing, Nanjing Normal University, Nanjing, 210042 China; 3 Department of Sports Medicine and Adult Reconstructive Surgery, Drum Tower Hospital affiliated to Medical School of Nanjing University, Nanjing, China

## Abstract

In the field of bone defect repair, gradient porous scaffolds have received increased attention because they provide a better environment for promoting tissue regeneration. In this study, we propose an effective method to generate bionic porous scaffolds based on the TPMS (triply periodic minimal surface) and SF (sigmoid function) methods. First, cortical bone morphological features (e.g., pore size and distribution) were determined for several regions of a rabbit femoral bone by analyzing CT-scans. A finite element method was used to evaluate the mechanical properties of the bone at these respective areas. These results were used to place different TPMS substructures into one scaffold domain with smooth transitions. The geometrical parameters of the scaffolds were optimized to match the elastic properties of a human bone. With this proposed method, a functional gradient porous scaffold could be designed and produced by an additive manufacturing method.

## Introduction

The goal of tissue engineering is to promote the regeneration of tissues by combining a scaffold, cells and active molecules. As such, it is important to fully understand the structure and functionality of bone under normal conditions, as the goal of bone tissue engineering is to repair or replace damaged bone tissue with the original organization and function after recovery^1–4^. Bone tissue engineering’s basic goal is to implant a three-dimensional composite scaffold constructed from biological material and cells into a lesion site. In the *in vivo* environment, the cells will undertake a variety of physiological activities around the scaffold to repair the lesion.

One of the key parameters in the manufacture of tissue engineering scaffolds is the creation of a porous structure inside the scaffold. These internal pores directly affect the growth state of the cells^5,6^. In general, the scaffold provides a biological microenvironment that stimulates cell growth behavior through cell-scaffold and cell-cell interactions, such as adhesion, migration, proliferation, differentiation, maintenance and death^7,8^. Initial support is necessary and can be provided to the tissue culture by the scaffold *in vitro* or *in vivo*. Additionally, cell adhesion and a controllable regular distribution of cells must be ensured. An ideal tissue scaffold should have the following characteristics^9^: (1) the scaffold should contain a porous structure with high porosity to be conducive to cell growth, nutrient transport and metabolite discharge. (2) The scaffold should be biocompatible and biodegradable at rates that are compatible with tissue growth, and the degradation products must have no effect on tissue metabolism. (3) The surface micro-topography must promote cell adhesion, proliferation and differentiation. (4) The scaffold must have mechanical properties similar to those of the surrounding tissue.

Bone tissue is an anisotropic, complex structure, and ideal internal pore structure has been constructed by natural selection and evolution. Bone tissue has not only excellent mechanical properties and permeability but also a variety of pore morphologies that are adapted to the growth needs of cells^10–12^. The internal structure of the bone tissue has a specific porosity and mechanical strength, and the internal communication pore allows the transport of nutrients from the outside to the inside of the system, which creates a favorable biological environment for cell growth^13,14^. The anisotropic pore structure within the internal scaffold may guide the development of bone growth factor in the direction of the porosity and mechanical gradient, which would be helpful for controlling stem cell differentiation and promoting tissue function. Different pore structures can affect the movement and metabolism of bone cells, which can act as a physiological mechanism for controlling pore cell behavior in an extracellular environmental.

When studying models for porous bone tissue, cancellous bone is typically replaced by a homogeneous porous structure. The morphological characteristics of the cancellous bone cannot be truly replicated, meaning that the functional parts of the designed and manufactured bone tissue are inadequate, and the synthetic treatment cannot perfectly replace the bone defect site^15^. In the manufacture of porous scaffolds, some of the traditional, hand-made techniques, such as salt filtration, particle-induced porosity, gas foaming, and fiber bonding, can only achieve rough control of some parameters including porosity and pore size. The porosity, connectivity and uniformity of the microporous structure in the scaffold cannot be precisely designed before the manufacturing process, and it is difficult to accurately control the pore growth during the manufacturing process^16^. It is often difficult to meet the manufacturing requirements of bionic bone tissue because the pore characteristics of the bone scaffold cannot effectively be controlled (including pore distribution, pore size, and shape). Recently developed three-dimensional printing technology (such as selective laser sintering, light-curing three-dimensional printing, and hot melt extrusion technology) has solved this problem and has shown great advantages in the preparation of personalized porous bone scaffolds^17–19^. This technique decomposes a 3D model into a series of layers; next, using point-to-face and surface-to-body stacking, the macroscopic structure of the bone scaffold is obtained. The micro-topology of the scaffold can also be controlled to a certain extent.

Currently, there are still many challenges facing the design and construction of bionic porous scaffolds for three-dimensional printing, such as how to facilitate the on-demand design of porous structures with bionic performance and what the designs should be based on^20–24^. In this paper, the 3D morphological features of a macroscopic bone and the micro-morphological features of a trabecular bone were observed by reverse-modeling femoral condylar micro-CT data from New Zealand rabbits. The 3D structures of the proximal femur and the trabecular bone in the femoral head region were established and analyzed. The internal pore structure of the bone was quantitatively analyzed to assign the structural regularity of different regions of bone. Additionally, using the TPMS modeling method^25–27^, the porosity and pore size distribution of the scaffold could be controlled by adjusting the pore size and morphological controls in the implicit function. Using this method, the bionic porous bone scaffolds were accurately constructed with the desired pore parameters. Finally, 3D printing technology was used to accurately manufacture the required porous structures.

## Materials and Methods

All methods in this study were carried out in accordance with relevant guidelines and regulations. All experimental protocols in this study were approved by the committee of Drum Tower Hospital affiliated to Medical School of Nanjing University.

### Micro-CT data reconstruction

**Figure 1:**
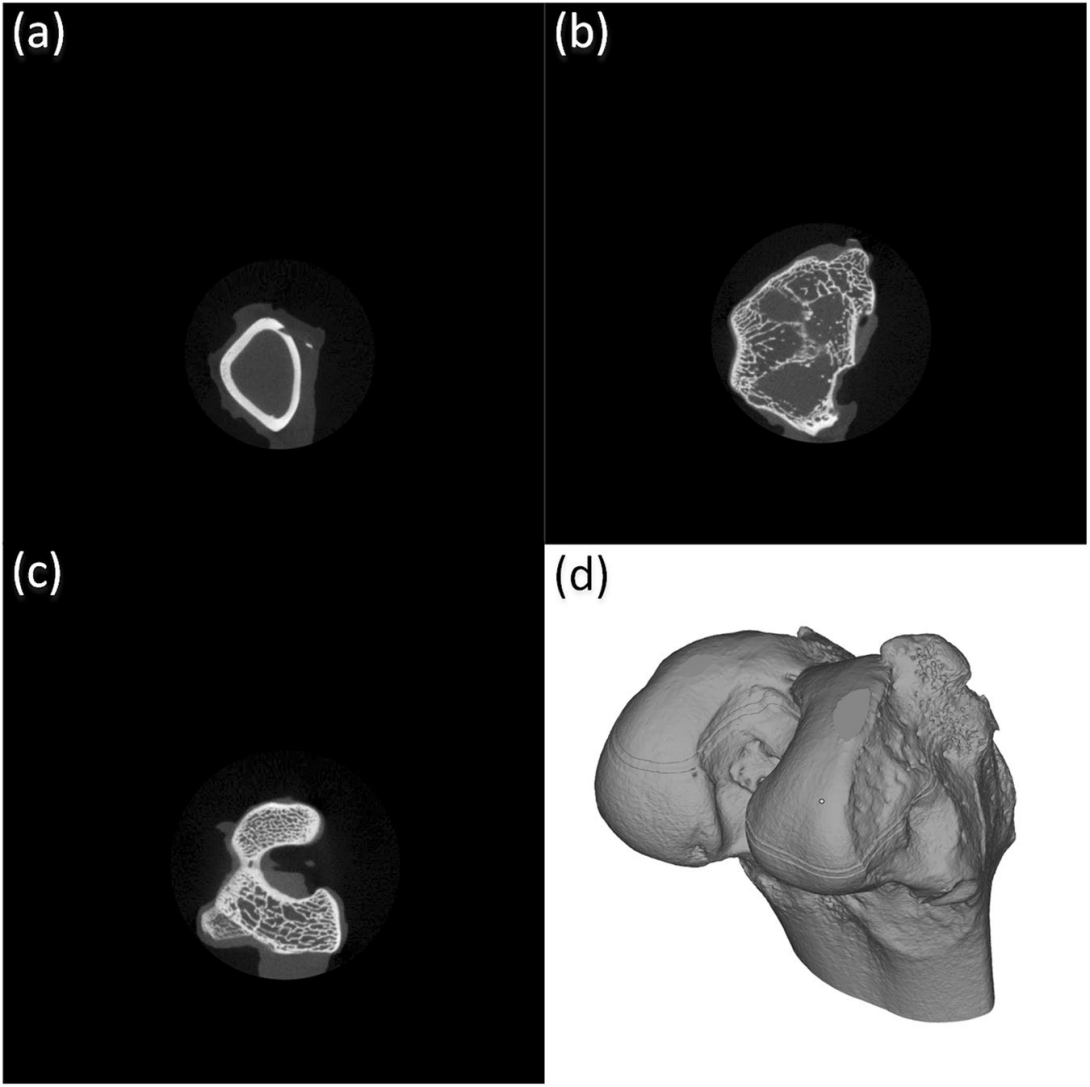
3D reconstruction of the femoral condyle bone of a New Zealand rabbit by CT images, (**a**) CT image of the 106^th^ layer, (**b**) CT image of the 216^th^ layer, (**c**) the image of the 406^th^ layer, (**d**) femoral condyle bone as modeled by CT images.

In this paper, the femoral condyle of New Zealand rabbits (a sacrificed 5-month-old male New Zealand white rabbit with a weight of 2.6 kg) was chosen as the research object. The muscular and connective tissues were removed, and the geometric integrity of the samples was maintained. The bone samples were scanned using a micro-CT machine (Skyscan 1076 micro-CT scanner, Bruker MicroCT, Belgium) to obtain a stratified 2D image. The acquired CT diagram is in two-dimensional grayscale, and contains the basic information about the three-dimensional solid and the internal pore morphology of the sample. For this series of CT images, medical image processing software (Mimics, Materialise, Belgium) was used to build a three-dimensional model of the bone scaffold, as shown in Fig. 1. The bone shape and internal pore distribution were characterized by analyzing the 3D model.

### Acquisition of physical characteristics

**Figure 2:**
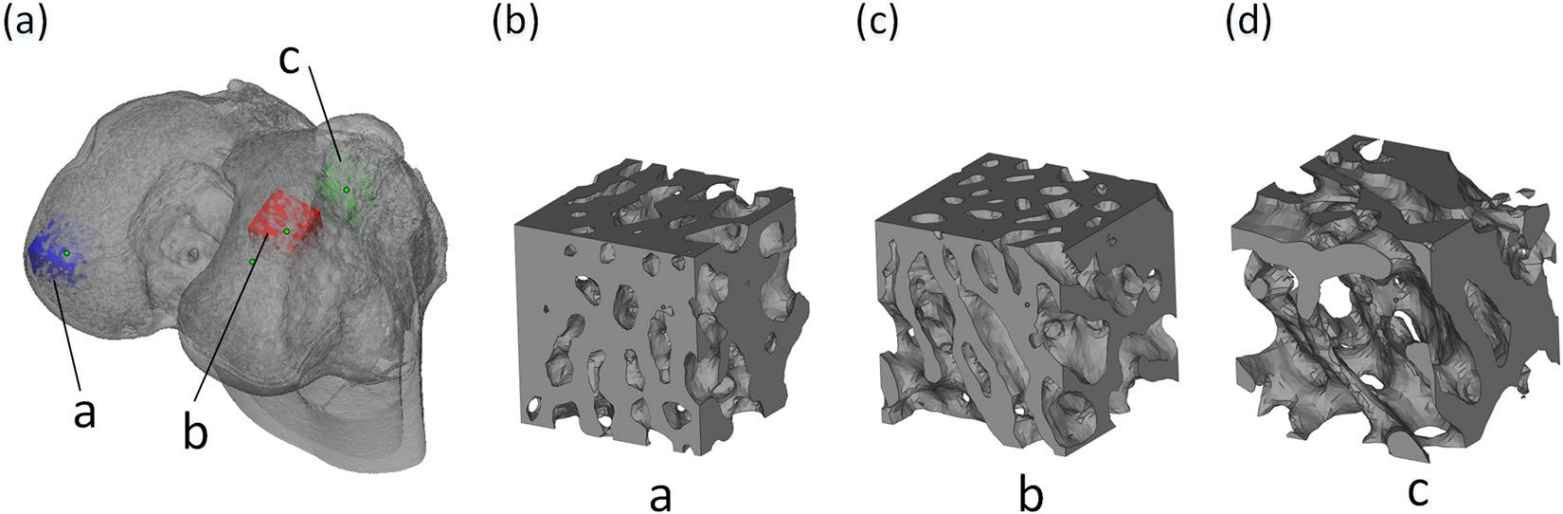
Morphological characteristics of bone units in different regions, (**a**) femoral condyle bone model, (**b**) top region of the femoral condyle bone, (**c**) side region of the femoral condyle bone, (**d**) inner region of the femoral condyle bone.

Three bone units were extracted from different parts of the femoral condyle bone, which was acquired in Section 2.1. Bone cells with different morphological characteristics were obtained as shown in Fig. 2, which also shows the three-dimensional morphological characteristics of a typical trabecular trapezoid in three regions. The structural characteristics of the bones show that in the a, b areas along the main growth direction, the trabecular bone is mostly made up of thick, plate-like bone trabeculae; along the other two directions, the trabecular bone is relatively thin; and most of the bone consists of rod-shaped trabeculae. The c region is composed of bony trabeculae with numerous confused and discontinuous growth directions. Therefore, it is difficult to clearly identify the main growth direction of the trabeculae from the morphological features of the trabecular bone in this region. However, the region’s plate-like structure is considerably thinner than the outside area, and the pores are considerably more uniform than the outside.

To describe the morphological characteristics of different regions of the trabecular bone, we selected four common trabecular morphology parameters, including TS (Trabecular Separation), TT (Trabecular Thickness), SAD (surface area density) and SMI (structure model index). The morphological parameters of the trabecular bone are defined as follows:

- TS: the mean diameter of the fitting circle between the trabecular bone and the medullary cavity by fitting the circle.
- TT: the average thickness of the trabecular bone in the specified area.
- SAD: the total surface area of the bone trabeculae per unit of bone volume is mainly used to evaluate the number of bone cells attached to the bone surface.
- SMI: Defines the extent to which the trabecular bone looks similar to a plate or rod shape. The SMI of the pure plate trabecular bone was 0 and the SMI of the pure rod shape trabecular bone was 3.

### Acquisition of mechanical characteristics

The three-dimensional morphology and structural characteristics of the trabecular bone show complex heterogeneity and anisotropy at the macro-level. Analyzing and studying the characteristics of bone mechanics in the construction of the bone tissue scaffolds is important to provide a reference for the synthetic scaffold. Each respective bone unit was analyzed along three orthogonal directions by the finite element software, and the correlations between different regions, different directions, different trabecular bone morphological features and bone mechanical properties were observed. The non-uniformity and anisotropy of the bone material at the tissue level are analyzed and the mechanical characteristics of the femoral head mechanical are discussed. In this paper, finite element analysis software (ABAQUS, Dassault Systems, USA) was used to load and analyze all the finite element models of the bones. A four-point tetrahedron was used to mesh the model, and the mesh cell size did not exceed 50 μm. The number of body units in the model ranges from 261480 to 363834. Since the gray value of the Micro-CT image has a linear relationship with the sample density, the bone density can be calculated from the gray value of a hydroxyapatite model image of known density. To reduce the calculation scale and improve the calculation efficiency, an elastic modulus (close to air) of 0.01 MPa is assigned to all the elements that have an elastic modulus of 5 MPa or less. The elastic modulus and the trabecular bone density formula are shown in Equation (1). The Poisson’s ratio is specified as 0.3.

1 $${\rm{E}}=\{\begin{array}{ll}6850\times {\rho }^{1.49} & \rho \le 1.68\\ 4239\times {\rho }^{2.39} & \rho  > 1.68\end{array}(MPa)$$

**Figure 3:**
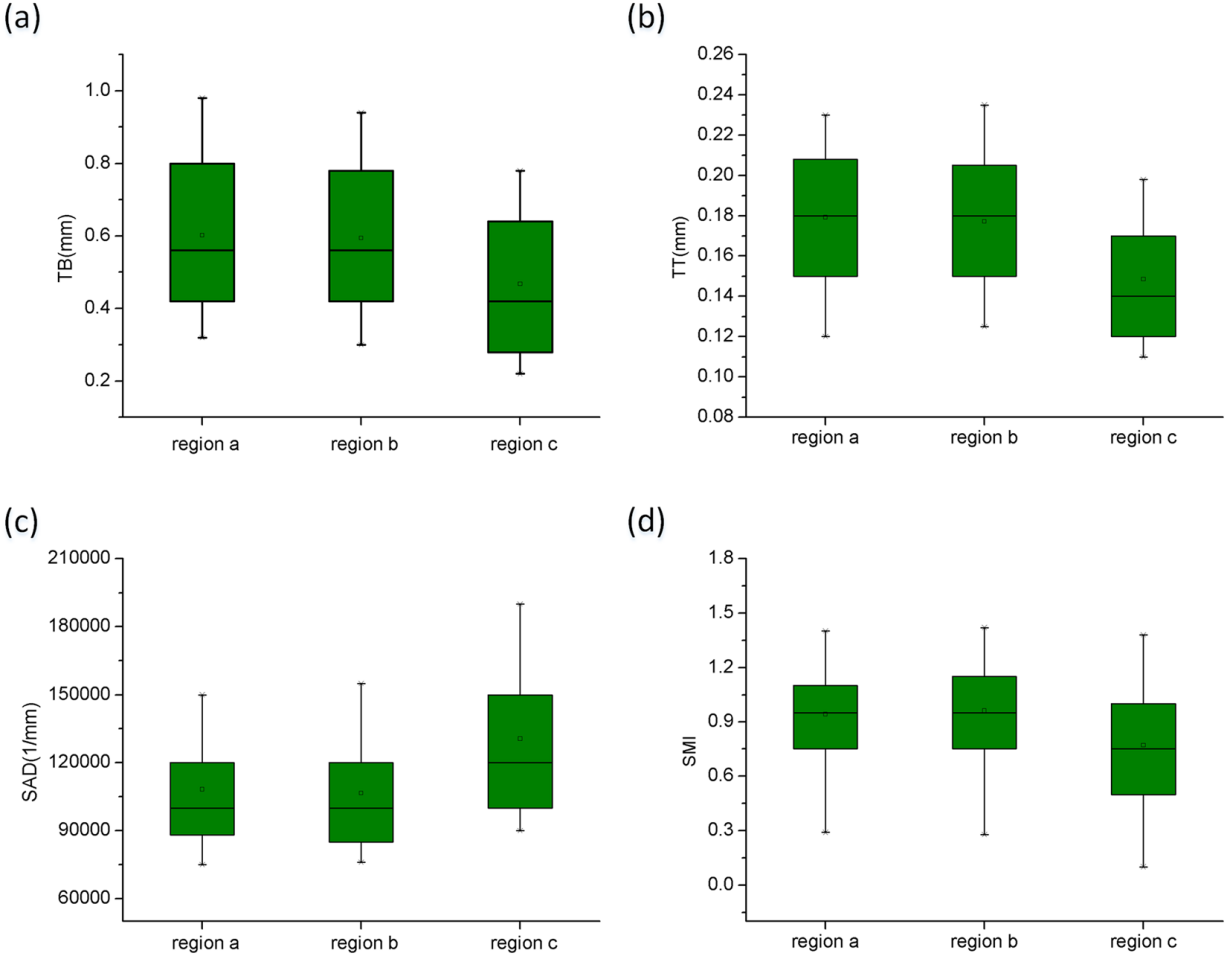
Finite element model used to apply boundary conditions along the three orthogonal directions. The pink arrow represents the position and direction of the displacement constraint (the constraint size is 1%), and the blue mark shows that the surface of the mark is completely constrained.

Each bone cell block is subjected to the same displacement constraint in three orthogonal directions, and a pressure of 1% of the compressive strain is applied perpendicularly to one surface of the bone model in the direction 1. Its relative surface is subjected to complete confinement, and it is applied to the microstructural analysis. The same boundary conditions are applied to the bone mass along direction 2 and direction 3, respectively. Figure 3 shows the finite element model of the top region of the bone, with boundary conditions applied in three orthogonal directions, respectively. The von Mises stress and the apparent elastic modulus of the bone along the loading direction can be obtained by analyzing the finite element analysis in three orthogonal directions.

In this study, a reverse micro-CT technique and finite element analysis were used to quantitatively analyze the morphological parameters and mechanical parameters of a trabecular bone in the internal area of the femoral head. By analyzing the measurements, we found that there were significant regional differences in the morphological parameters and mechanical parameters of the trabecular bone. These two parameters also have significant internal relationships. In the cancellous bone, the main bearing direction is mainly composed of plate-like trabeculae, and the non-main bearing direction is mainly composed of rod-shaped trabeculae. This feature determines the anisotropic mechanical properties of the bone along different loading directions. The regional differences, anisotropic properties, and microstructural regional differences of the trabecular bone are important factors affecting bone performance. Our analysis of these parameters can be used as an important reference for bionic bone scaffold design.

### TPMS modeling method

TPMS are minimal surface models that exhibit periodicity in three independent directions in three-dimensional space. The surface has a mean curvature of zero, and can be extended indefinitely in three periodic directions. It can provide a concise description for many physical structures, such as silicates and soluble colloids. As a pore forming unit, TPMS can realize the digital representation of a porous structure.

There are many ways to generate TPMS coordinates, which have an exact parameterized form defined by the Weierstrass formula, as shown in Equation (2):

2 $$\{\begin{array}{l}x=Re{\int }_{\omega 0}^{\omega 1}{e}^{i\theta }(1-{\omega }^{2})R(\omega )d\omega \\ y=Re{\int }_{\omega 0}^{\omega 1}{e}^{i\theta }(1+{\omega }^{2})R(\omega )d\omega \\ z=Re{\int }_{\omega 0}^{\omega 1}{e}^{i\theta }(2\omega )R(\omega )d\omega \end{array}$$

where ω represents a complex variable, θ is an angle called Bonnet, and *R* (*ω*) represents a function that varies with different surfaces. For P, D and G surface of TPMS, *R* (*ω*) can be expressed by the Equation (3):

3 $$R(\omega )=\frac{1}{\sqrt{1-14{\omega }^{4}+{\omega }^{8}}}$$

Compared with parametric TPMS, the periodic surface in approximate TPMS is generally defined, and can be expressed as shown in Equation (4):

4 $$\varphi (r)=\sum _{k=1}^{K}{A}_{k}cos[\frac{2\pi ({h}_{k}\cdot r)}{{\lambda }_{k}}+{p}_{k}]=C$$

where γ represents position vectors of Euclidean space, *A*_*k*_ represents the amplitude factor, *h*_*k*_ is the k^th^ grid vector in the reciprocal space, *λ*_*k*_ represents the periodic wavelength, *p*_*k*_ represents the phase offset, and C is a constant.

Approximate TPMS is formulated by an implicit function, and each point on its surface has a constant value. Because the surface is processed using this characteristic, it is called an isosurface. Some approximate TPMS are listed in the Table 1, where the standard values are $\varphi {(r)}_{0}=0$, X = 2πx, Y = 2πy, and Z = 2πz.

**Table 1:** Some simple TPMS models constructed by trigonometric function.

TPMS	periodic trigonometric function
P	*ϕ* (*r*) = cos(*X*) + cos(*Y*) + cos(*Z*) = 0
D	*ϕ* (*r*) = cos(*X*) cos(*Y*) cos(*Z*) − sin(*X*) sin(*Y*) sin(*Z*) = 0
G	*ϕ* (*r*) = sin(*X*) cos(*Y*) + sin(*Z*) cos(*X*) + sin(*Y*) cos(*Z*) = 0

For an unclosed implicit surface defined by an implicit function, one or more intersecting surfaces need to be defined in order to structure a closed surface. The main challenge in structuring curved surfaces is solving the intersection line of the curved surface in three-dimensional space. For the intersection operation of both an implicit surface and parametric surface, a plane algebraic surface equation can be obtained by plugging the parametric function into the implicit function, and the problem can then be solving by plane algebra theory. To solve the intersection operation of implicit surfaces, one of the surfaces must be parameterized by the method mentioned above.

With the parameter domain in hand, the parametric surface can be constructed and triangulated by directly calculating the position of the point on the surface. Because the implicit surface does not have this parameter, it is hard to implement a similar transformation.

A MC (Marching Cubes) method was employed for the triangulation of the implicit surface. The basic principle of this method is that the intersection points of the isosurface and the hexahedron edge are connected in a specific way, and the new isosurface is approximated to represent the implicit surface.

### Bionic bone construction

As shown in Equation (4), the parameters involved in the surface modeling are *A*_*k*_, *h*_*k*_, *λ*_*k*_, *p*_*k*_ and the standard value $\varphi {(r)}_{0}=C$. The control of the TPMS surface modeling is adjusted by the trigonometric parameters in the function. The TPMS surface type is mainly controlled by the four parameters *A*_*k*_, *h*_*k*_, *λ*_*k*_, and *p*_*k*_. The required TPMS surface can be constituted by assigning different values. In this paper, a Tubular-G (TG) surface is chosen as the bionic bone scaffold unit due to its bony structure. The distribution characteristics of the TGa, TGb, TGc structures should correspond to region a, region b and region c, respectively (section 2.2).

In Section 2, the extraction position of the bone unit in regions a and b belongs to the near surface of the bone, and its morphological structure is similar. The extracted natural bone simulation shows that the mechanical properties of units a and b are similar, except for the direction of the load. Therefore, the same structure is used for the bionic design of the model units TGa and TGb. The finite element analysis of the TG surface element is carried out under the same simulation conditions, and the simulation results are compared with the bone tissue simulation results (Section 2). By constantly adjusting the structural parameters, the simulation results of the TG model are forced to close to the natural bone tissue.

Since the bone scaffold is a complex structure that consists of different structural regions, it is necessary to splice the different units together. In this paper, a sigmoid function is employed to fuse the multi-unit features. A special function is needed as a bridge to incorporate the different scaffolds into a single model. To avoid mutation of the model, a continuous function can be used to recognize the discontinuous change of structure in the model. The whole structure can be defined as shown in Equation (5).

5 $${\varphi }_{sf}(x,\,y,\,z)=\alpha (x,\,y,\,z){\varphi }_{1}(x,\,y,\,z)+(1-\alpha (x,\,y,\,z)){\varphi }_{2}(x,\,y,\,z)$$

where *ϕ*_*sf*_ represents the function of the final fused structure and *ϕ*_1_ and *ϕ*_2_ represent the respective different units in the structure. The formula $\alpha (x,\,y,\,z)$ is a weight function that describes a monotonic transformation from [0, 1]. The weight function is defined as shown in Equation (6).

6 $$\alpha (x,\,y,\,z)+\frac{1}{1+{e}^{-kG(x,y,z)}}$$

where $G(x,\,y,\,z)$ is a continuous function, the transition boundary is defined by $G(x,\,y,\,z)=0$, which is intended to intersect two different TPMS scaffold units. The transition gradient can be adjusted by changing the parameter *k*. In this paper, $\alpha (x,\,y,\,z)={x}^{2}+{y}^{2}+{z}^{2}-C$ by substituting Equations (5 and 6) into formula (5).

## Results and Discussion

### Physical characteristics

**Figure 4:**
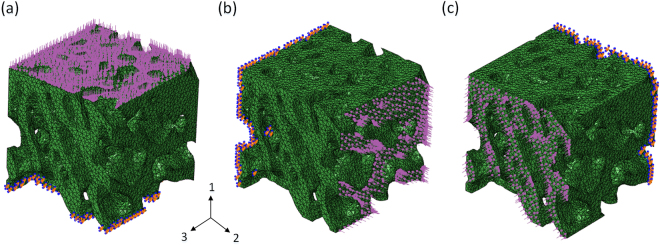
Statistical table of the morphological parameters of the trabecular bone in different regions of the femoral head.

The significant differences in the trabecular bone morphological parameters for different regions of the femoral condyle bone are shown in Fig. 4. From the medial region of the femoral condyle bone to the lateral region, the TT shows a significant increase, whereas SAD decreases. According to the functional adaptation and daily load of the bone, the cancellous bone tissue mainly maintains the strength of the trabecular bone by increasing its thickness. According to the results, the thickness of the trabecular bone is significantly smaller in the medial region than in the lateral region. Simultaneously, the porosity is significantly larger in the medial region than in the lateral region. This finding suggests that the bone density of the cancellous bone in the medial region of the femoral condyle is smaller. SMI represents the contrast between the amount of the trabecular bone that is composed of plate-like structures in the cancellous bone region and the amount of the trabecular bone that is primarily composed of rod-like structures. Figure 2 clearly shows that in the medial region, the morphology of the trabecular bone structure gradually changes from the multi-plated lateral trabecular bone to the multi-rod structure.

### Mechanical properties

**Figure 5:**
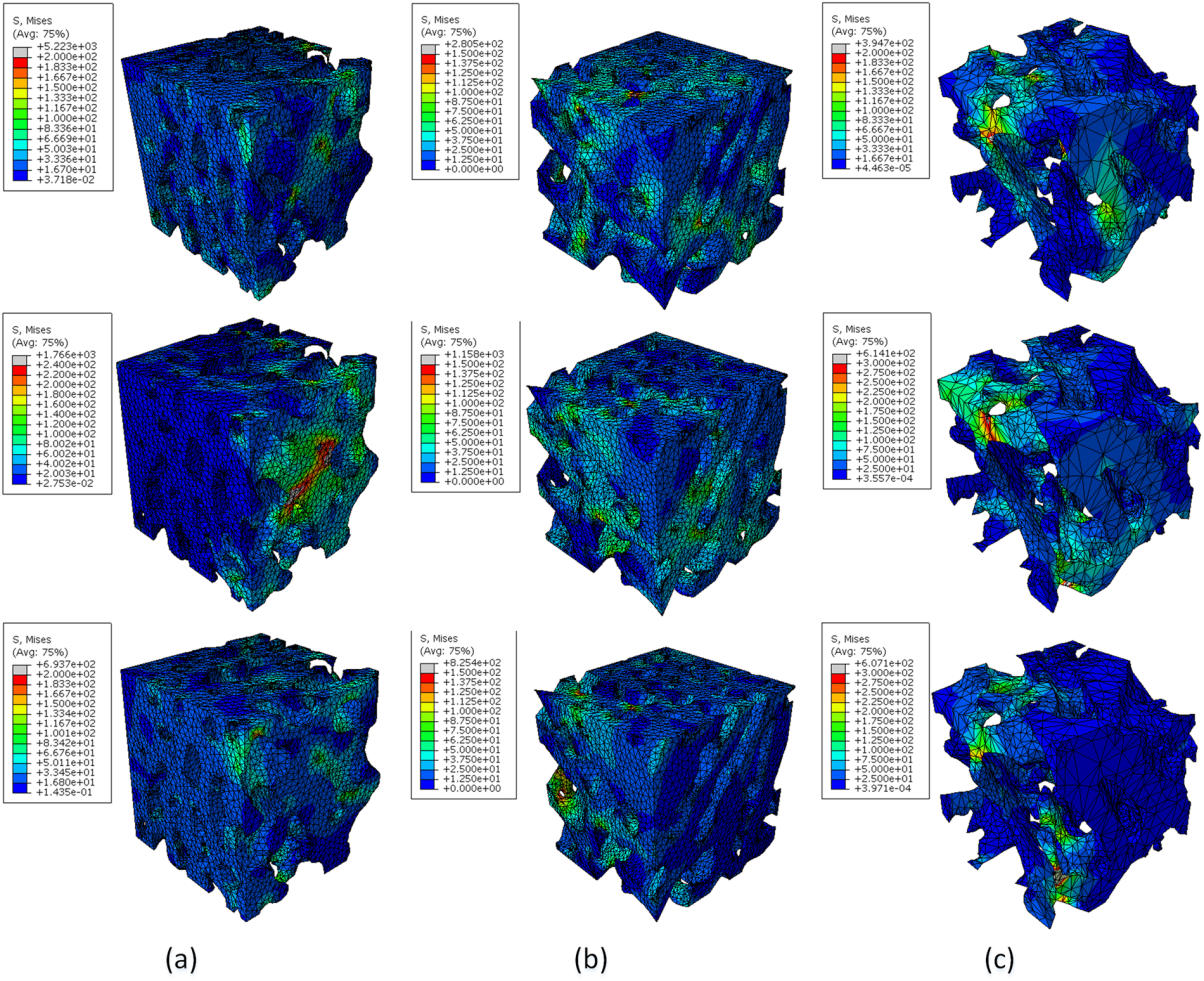
Von Mises stress distributions of a typical bone sample in different regions of the bone.

Figure 5 shows the von Mises stress cloud diagram of a typical bone in different regions of the femoral head for three orthogonal directions. The figure clearly shows the various regions of the bone structurally correspond to the direction of the load applied in a particular situation: a, b regions are composed of plate-like trabeculae, and region a grows in direction 2, while region b grows in direction 1. So, the bulk cancellous bone strength of a and b is higher, and their average von Mises stress is 7.8 MPa. The plate-like trabeculae are thin in region c, and the regional distribution of cancellous bone in region c is more uniform than in the lateral region, and the mean value of stress is 4.48 MPa.

In region b, the apparent elastic modulus and the mean von Mises stress in direction 1 are significantly larger than those in direction 2 and direction 3, though the mechanical properties of direction 2 and direction 3 are not significantly different. The mechanical parameters calculated in direction 1 show that the apparent elastic modulus and von Mises stress in the outer region are significantly larger than those in the medial region, indicating that the lateral region has the strongest compressive ability. There was no significant difference in the mechanical parameters calculated from the non-growth axis along the three directions. The trabecular bone in the b region is close to direction 2 and direction 3. For region b, direction 1 is the main bearing direction for the daily standing and daily gait processes, and direction 2 and direction 3 are not the primary bearing directions. In the same analysis versus region a, direction 2 is the main load direction for the daily gait process.

Significant regional differences are seen not only for the proximal morphological parameters of the bone but also the mechanical parameters calculated from the finite element analysis show significant anisotropy and regional differences. According to the von Mises stress distribution results, the strength of the cancellous bone mass in the transverse direction is much smaller than in the axial direction for all regions, and the average von Mises stress is less than 50% of the axial mean stress. In addition, the lateral mean von Mises stress of the cancellous bone did not show significant regional differences for the three regions, indicating that the laterally grown trabecular bone was essentially consistent in all regions. The microstructural characteristics of the trabecular bone in the femur showed significant anisotropy. The mechanical properties of the cancellous bone were also affected by the morphological structure of the cancellous bone, and the mechanical properties of the cancellous bone also showed significant regional differences.

### Typical TPMS models

After extracting the isosurface by the Marching Cube method, the corresponding STL model can be conveniently extracted. The STL model contains the triangular surface information for the external surface of the object. Moreover, the quality of the triangular patch has no real influence on the extraction and expression of the STL model. Therefore, this method has obvious advantages for porous structures or porous implants that need to be made by an additive manufacturing process.

**Figure 6:**
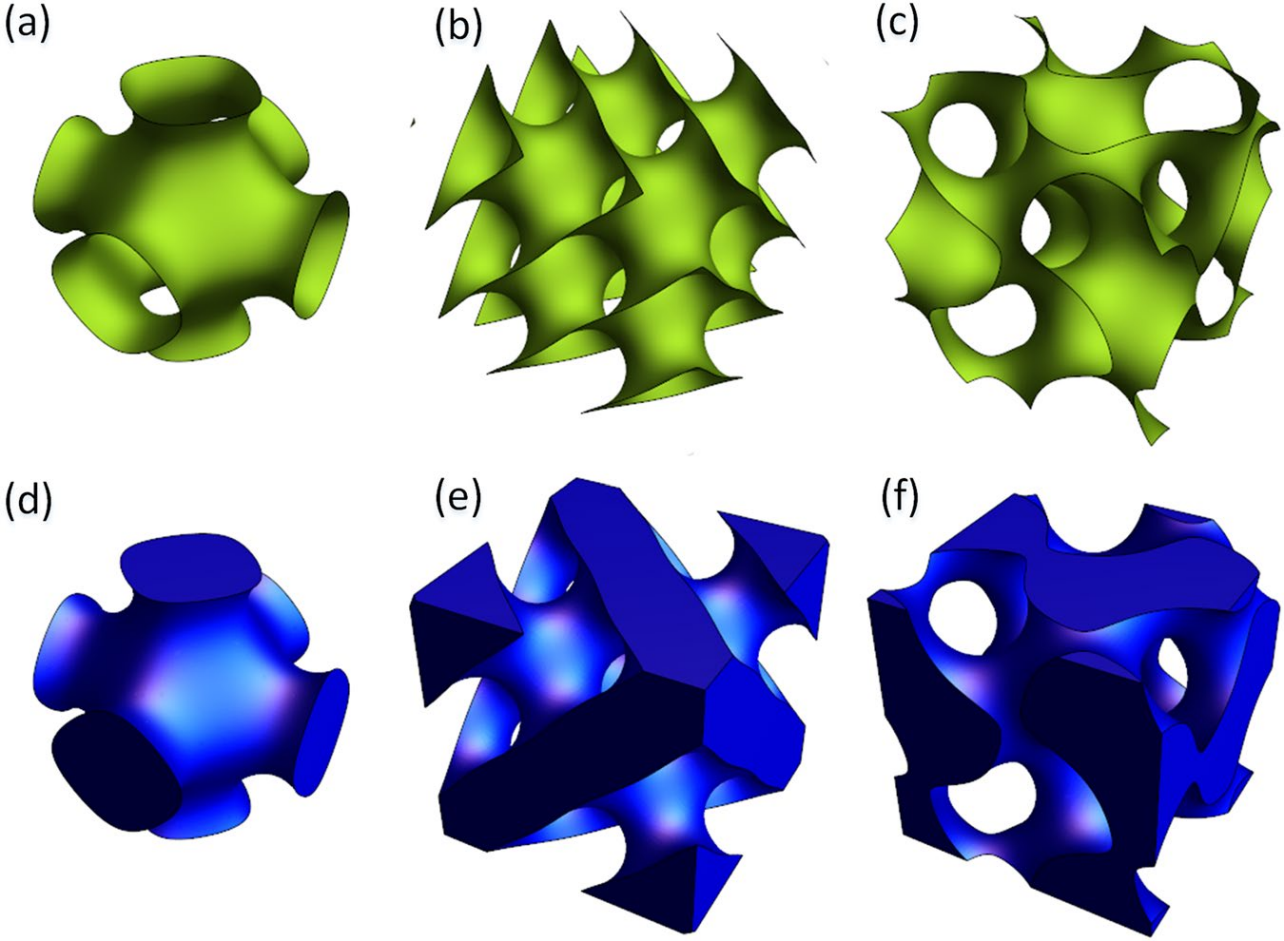
Triangulation of a non-closed implicit surface: (**a**) P surface, (**b**) D surface, (**c**) G surface; triangulation of typical closed implicit surface: (**d**) P surface, (**e**) D surface, (**f**) G surface.

The typical TPMS P, D, and G surfaces are triangulated and visualized separately, as shown in Fig. 6.

### Bionic bone scaffold

**Figure 7:**
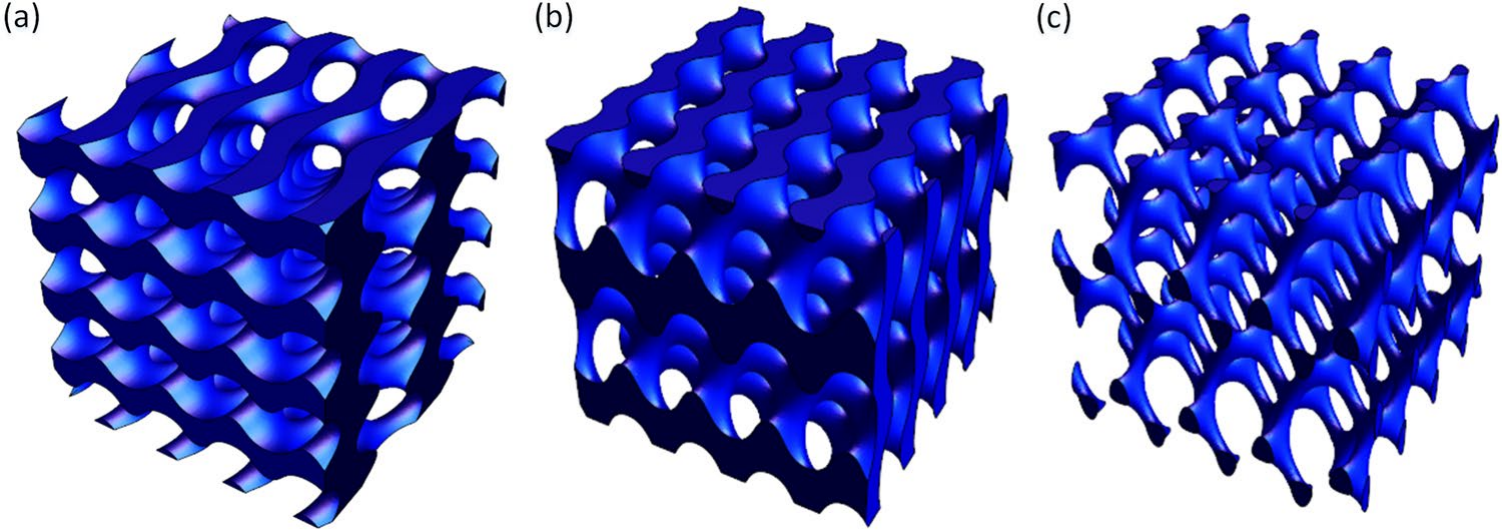
Bionic porous scaffold model structure, (**a**) TGa scaffold, (**b**)TGb scaffold, (**c**)TGc scaffold.

A model unit of a bionic bone scaffold with different cell structures was constructed, as shown in Fig. 7. The TPMS function for TGa and TGb is shown in Equation (7), and the TPMS function of TGc is shown in Equation (8).

7 $$\begin{array}{rcl}T{G}_{a,b} & = & 20(\cos (x)\sin (y)+\,\cos (y)\sin (z)+\,\cos (z)\sin (x))-0.5(\cos (2x)\cos (2y)\\  &  & +\,\cos (2y)\cos (2z)+\,\cos (2z)\cos (2x))-4\end{array}$$

8 $$\begin{array}{rcl}T{G}_{c} & = & 10(\cos (x)\sin (y)+\,\cos (y)\sin (z)+\,\cos (z)\sin (x))-2(\cos (2x)\cos (2y)\\  &  & +\,\cos (2y)\cos (2z)+\,\cos (2z)\cos (2x))-12\end{array}$$

**Figure 8:**
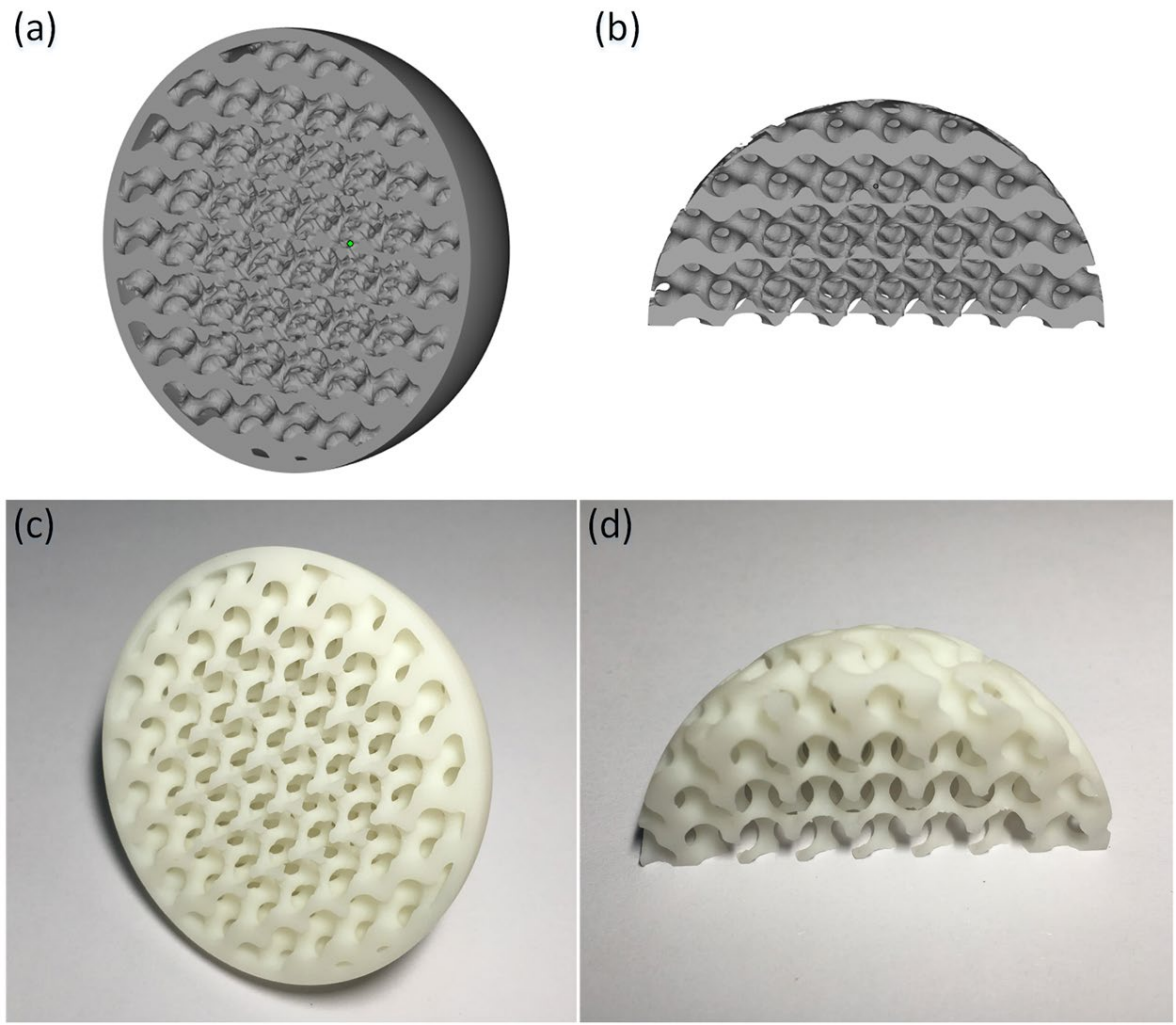
Modeling and fabrication of a porous bionic scaffold: (**a**,**b**) designed model with gradient structure; (**c**,**d**) printed bionic porous scaffold sample.

The different units are spliced together using the MC method, then the fused porous structure model with a gradient structure can be obtained, as shown in Fig. 8(a,b). The model designed scaffold was fabricated by 3D printing, as shown in Fig. 8 (c,d). The results show that the bionic structure is well-represented by the method proposed in this study.

## Conclusions

In this paper, binarization processing of scanned micro-CT images was used to obtain the morphological features and distribution characteristics of pores in a skeleton. According to the analysis of the bone units, a porous scaffold with controllable pore distribution can be established using a TPMS modeling method. The model is analyzed using FEA software and compared to the FEA results of reversing the bone units. By adjusting the parameters of the TPMS functions, the mechanical properties of the model are adjusted and forced to close to the reversed bone model. The structural characteristics and mechanical properties of the TPMS models are analyzed. The results show that the performance of the TPMS modeling scaffold is close to that of the reversed bone model, which can effectively be used as a gradient porous scaffold for bone tissue engineering.
